# Static Magnetic Field Increases Polyhydroxyalkanoates Biosynthesis in *Haloferax mediterranei*: Parameter Optimization and Mechanistic Insights from Metabolomics

**DOI:** 10.3390/polym17091190

**Published:** 2025-04-27

**Authors:** Ze-Liang Gao, You-Wei Cui

**Affiliations:** National Engineering Laboratory for Advanced Municipal Wastewater Treatment and Reuse Technology, Faculty of Environment and Life, Beijing University of Technology, Beijing 100124, China; 020327g@emails.bjut.edu.cn

**Keywords:** polyhydroxyalkanoate synthesis, biodegradable materials, static magnetic field, magnetic parameter optimization, metabolomics analysis, halophilic archaea, circular bioeconomy

## Abstract

Polyhydroxyalkanoates (PHAs), as biosynthetic and biodegradable polymers, serve as alternatives to petroleum-based plastics, yet face critical cost barriers in large-scale production. While magnetic field (MF) stimulation enhances microbial activity, the optimal MF parameters and metabolic mechanisms for PHA biosynthesis remain unexplored. This study optimized magnetic field parameters to increase PHA biosynthesis in *Haloferax mediterranei*. A custom-engineered electromagnetic system identified 110 mT of static magnetic field (SMF) as the optimal level for biosynthesis, reaching 77.97 mg/(L·h) PHA volumetric productivity. A pulsed magnetic field caused oxidative stress and impaired substrate uptake despite increasing PHA synthesis. Prolonged SMF exposure (72 h) maximized PHA productivity, while 48 h of exposure attained 90% efficiency. Metabolomics revealed that SMF-driven carbon flux redirection via regulated butanoate metabolism led to a 2.10-fold increase in (R)-3-hydroxybutanoyl-CoA), while downregulating acetoacetate (0.51-fold) and suppressing PHA degradation (0.15-fold). This study pioneers the first application of metabolomics in archaea to decode SMF-induced metabolic rewiring in *Haloferax mediterranei*. Our findings establish SMF as a scalable bioenhancement tool, offering sustainable solutions for the circular bioeconomy.

## 1. Introduction

The extensive use of petroleum-based plastics has contributed to an ecological crisis, including depletion of non-renewable fossil fuels, global white pollution, and microplastic contamination. These collectively threaten human health and have socioeconomic impacts [1]. Polyhydroxyalkanoates (PHA) are a promising biodegradable alternative, experiencing complete microbial degradation within two months [2,3]; however, their commercialization is constrained by production costs that are 10-fold higher than polypropylene (2–6.5 vs. 0.2 USD/kg) [4,5]. This cost disparity is mainly due to the fermentation substrates. This makes halophilic archaea such as *Haloferax mediterranei* (*H. mediterranei*) particularly attractive due to their ability to synthesize PHA using low-cost carbon sources [6,7]. However, the industrial application of *H. mediterranei* faces a critical problem: PHA volumetric productivity is low due to the slower growth rate. This prevents rapid, high-cell-density cultivation [8]. Addressing this challenge requires fundamental improvements in metabolic activity to simultaneously increase cellular PHA levels and biomass concentration.

Recent advances in magnetic field (MF) biotechnology have revealed a potential solution [9,10]. Static magnetic fields (SMFs) have increased microbial effects across species [11], including increased substrate utilization in *Rhodococcus erythropolis* [12], improved colony-forming capacity in *Escherichia coli* [13], and altered fungal colony size and hyphal pigmentation [14]. The optimal intensity of the SMFs varies widely among different microorganisms. For instance, endothelial cells exposed to 120 µT SMF for 2 days exhibited 40% increased growth [15], while *Salmonella* showed enhanced antioxidant capacity under 200 mT SMF [16]. The positive effects occur most often at SMF intensities below 50 mT [17,18,19]. For *H. mediterranei*, preliminary studies indicate that an SMF (50 mT at 30 wt% salinity) increases the PHA accumulation by 8% through SOD-mediated oxidative stress mitigation and glycine betaine (an osmoprotectant) synthesis [20], while 110 mT SMF alters intracellular carbon source allocation, redirecting carbon flux from the TCA cycle toward PHA precursor synthesis, thereby enhancing PHA productivity by 27% [21]. However, there are gaps in the SMF research. First, studies focus on intensity parameters but have not considered field type and temporal application strategies. Second, most studies depend on permanent magnets, creating spatially heterogeneous MFs with rapid decays in intensity. Third, omics-level analyses of bioeffects on *H. mediterranei* have gradually emerged, but there is a significant gap in mechanistic descriptions using metabolomics approaches.

To address these gaps. this study developed a custom-engineered electromagnetic system capable of generating homogeneous SMF. One-factor-at-a-time (OFAT) experimentation was used to systematically investigate the effects of three key parameters on the biomass accumulation and PHA volumetric productivity in *H. mediterranei*. The three parameters included MF intensities (105/110/115-mT), MF types (static and pulsed), and varied MF exposure durations (0/24/48/72-h). This multi-parametric optimization strategy establishes a new paradigm for increasing bioplastic production through bio-magnetic engineering. In addition, the research pioneered metabolomics analysis to decode SMF-induced metabolic rewiring in *H. mediterranei*. This fills a critical knowledge gap concerning the magnetobiological mechanisms governing PHA biosynthesis.

## 2. Materials and Methods

### 2.1. Strain Cultivation and Fermentation Method

The *H. mediterranei* used in this experiment was purchased from the China General Microbiological Culture Collection Center (Chaoyang District, Beijing, China; deposit accession number: CGMCC 1.3716). The strains were grown in a solid medium containing 7 g/L casamino acids, 10 g/L yeast extract, 20 g/L MgSO_4_·7H_2_O, 3 g/L sodium citrate, 2 g/L KCl, 10 mg/L FeSO_4_, 200 g/L NaCl, and 25 g/L agar (pH 7.2). They were incubated at 37 °C for 72 h and stored at 4 °C. Then, strains on the solid medium were inoculated into the same components of the liquid medium (without agar) in a 250 mL laboratory flask with a working volume of 50 mL; the strains were held at 37 °C at a rotational speed of 120 rpm for 60 h. Finally, a 50 mL sample of pre-inoculum was transferred to a bioreactor containing 1.0 L of fermentation broth (0.55 g/L CaCl_2_·2H_2_O, 13.00 g/L MgCl_2_·6H_2_O, 20.00 g/L MgSO_4_·7H_2_O, 0.25 g/L NaHCO_3_, 0.48 g/L KH_2_PO_4_, 0.50 g/L NaBr, 4.00 g/L KCl, 200.00 g/L NaCl, 10.00 g/L glucose, 0.50 g/L NH_4_Cl, 10.00 mg/L FeSO_4_, and 1.00 mL/L SL-6 trace elements; pH 7.2). The samples were incubated for 72 h at 37 °C.

### 2.2. Reactors Set-Up and SMF Exposure

**Figure 1:**
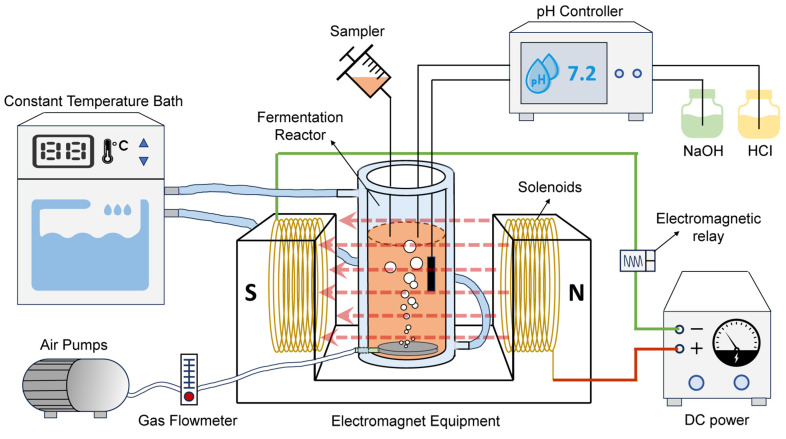
Graphical representation of experimental apparatus. The pink dotted arrows indicate the spatial distribution and orientation of the magnetic field generated within the electromagnetic device.

Figure 1 shows the study’s experimental procedures. The cylindrical bioreactor had a total volume of 2.0 L and an external double-glass structure. The inside of the double glass was filled with water. The temperature of the fermentation broth inside the reactor was controlled by a thermostatic circulating water bath (SDC-6, Xinzhi, Ningbo, China). Throughout fermentation, the pH was monitored using an online pH electrode (Multi 3420, WTW, Munich, Germany) and was maintained at a stable level using a pH auto-regulator (SC-100A, SiCheng, Changsha, China). The regulator was connected to two different pH-adjusting solutions, NaOH and HCl, each at a concentration of 1.00 mol/L. An aeration disc was set at the bottom of the reactor, connected to an air pump. The dissolved oxygen concentration in the reactor was maintained at about 2.0 mg/L using a gas flow meter.

An electromagnet containing multiple coils of copper wire was connected to a regulated DC power supply to form an SMF. Unlike permanent magnets, the intensity of the SMF generated inside the electromagnetic device has minimal fluctuations within the target spatial area (the pink dotted arrows in Figure 1); thus, it was considered to be a uniform SMF [22]. The SMF intensity could be adjusted by changing the magnitude of the current. The relationship between the current and the SMF intensity is shown in Supplementary Table S1. The SMF intensity was measured using a teslameter (WT10A, Weite Magnetics, Henan, China). This study applied an OFAT method to investigate individual magnetic parameters. In this experiment, the intensity of the MF was controlled by varying the current. The pulsed magnetic field (PMF) was executed by regularly changing the direction of the current (with a time interval of 20 min, i.e., 1/1200 Hz). An electromagnetic relay was used to automatically control the power supplies on a specific schedule, to experiment with different durations of SMF exposure time (0, 24, 48, and 72 h).

### 2.3. PHA Detection

The water samples were initially centrifuged at 10,350× *g* for 10 min to remove the supernatant. The precipitates were resuspended in distilled water, treated with 1 mL of 4% sodium hypochlorite for cell lysis, and vortexed to liberate intracellular PHA granules. After repeated centrifugation and two distilled-water washes, the PHA-containing precipitates were lyophilized. For solvent extraction, 20 mg of freeze-dried cell powder was mixed with 2 mL of 0.2% sodium benzoate (SB8610, Solarbio, Beijing, China) as an internal standard, followed by 2 mL of chloroform. The mixture was vortexed (60 s), thermally digested at 105 °C for 20 h, and subjected to liquid–liquid extraction with 1 mL deionized water. After centrifugation (12,000× *g*, 5 min, 4 °C), the lower chloroform phase was dehydrated with anhydrous sodium sulfate (0.5 g), centrifuged again, and 1 mL of the dried extract was transferred to GC vials.

PHA quantification was performed on a Thermo Trace1300 GC system (Thermo Fisher Scientific, Waltham, MA, USA) with a TG-1MS capillary column (30 m × 0.25 mm × 0.25 μm) and a flame ionization detector. Nitrogen with 99.99% purity served as the carrier gas at a constant flow rate of 1.0 mL/min, with a split ratio of 30:1. The injector and detector temperatures were set to 250 °C and 300 °C, respectively. The oven temperature program started at 80 °C for 2 min, ramped up to 240 °C at 20 °C/min, and was held for 2 min. Automated injections and data processing were conducted using Chromeleon 7.0 software. The ratio of the sample peak area to the internal standard peak area was used to calculate the PHA content.

### 2.4. Analytical Methods

During the experiments, chemical oxygen demand (COD) was monitored using standard methods [23]. The cell dry weight (CDW) was obtained by centrifuging 10 mL of fermentation liquid at 9000 rpm for 10 min; the resulting sample was then supplemented with distilled water to reach the original volume. This step was repeated three times to wash away the NaCl. The precipitates were then freeze-dried at −50 °C (FreeZone^®^, Labconco, KC, USA) until a constant weight was reached. The PHA synthetase activity was determined using the PHA synthetase ELISA detection kit protocol (ZhenKe, Shanghai, China). The superoxide dismutase (SOD) activity, malondialdehyde (MDA) content, and lactate dehydrogenase (LDH) concentration in *H. mediterranei* were determined using a detection kit protocol (BC0175, BC0020, BC0680; Solarbio, Beijing, China).

PHA content was expressed as a percentage of CDW by Equation (1). PHA volumetric productivity (mg PHA/(L·h)) was calculated according to Equations (2)–(3).

PHA content (wt%) = mg PHA/mg CDW × 100%(1)

X_PHA_ (mg/L) = PHA content × CDW (2)

(3) $$\mathrm{PHA}\;\mathrm{volumetric}\;\mathrm{productivity}\;\left(\mathrm{mg}\;\mathrm{PHA}/\left(L\cdot h\right)\right)=\frac{X_{PHA_{1}}-X_{PHA_{0}}}{T}$$

where $X_{PHA_{0}}$ and $X_{PHA_{1}}$ represent the PHA concentration (mg/L) at the beginning and the end of the fermentation phase, respectively. T denotes the total fermentation time (in hours).

The growth rate (*μ*) was calculated by differentiating the sigmoidal growth curve based on the Gompertz model. All experiments were repeated three times.

### 2.5. Metabolomics Analysis

The sample was transferred to a 2 mL EP tube with PBS. Then, the sample was centrifuged at 10,000× *g* for 15 min at 4 °C. A total of 110 mg of sample was weighed into an EP tube, and 1200 μL of extract solution (methanol/acetonitrile/water = 2:2:1, with isotopically-labelled internal standard mixture) was added. Then, the samples were vortexed for 30 s and sonicated for 10 min in ice-water bath. Then, the samples were incubated at −40 °C for 1 h and centrifuged at 12,000× *g* for 15 min at 4 °C. A total of 150 μL of the supernatant was transferred to a fresh glass vial for LC/MS analysis. Quality control (QC) samples were prepared by mixing equal aliquots of supernatants from all experimental samples, with the QC injection volume standardized to match that of individual analyses.

Chromatographic separation was completed using a ultra-high-performance liquid chromatography (UHPLC) system (Vanquish, Thermo Fisher Scientific, MA, USA) equipped with a BEH Amide column (2.1 mm × 100 mm, 1.7 μm). The mobile phase included the following: (A) a 25 mM ammonium acetate/ammonia hydroxide aqueous solution (pH 9.75); (B) acetonitrile, with a constant injection volume of 2 μL maintained at 4 °C. The eluted metabolites were analyzed using a Q Exactive HFX hybrid quadrupole-Orbitrap mass spectrometer (Thermo Fisher Scientific, MA, USA) operating in both positive and negative ionization modes [24].

Mass spectrometry parameters were optimized as follows: the sheath gas flow rate was 30 Arb; the Aux gas flow rate was 25 Arb; the capillary temperature was 350 °C; the spray voltage was ±3.6/3.2 kV (positive/negative mode); the full MS resolution was 120,000; and MS/MS resolution was 7500. Data-dependent acquisition (DDA) was performed using normalized collision energies of 10, 30, and 60 eV.

The raw data were converted to the mzXML format using ProteoWizard and processed with an in-house program, which was developed using R and based on XCMS [25], for peak detection, extraction, alignment, and integration. These data were processed as follows: (1) Peaks with >50% missingness in biological samples were filtered. (2) Total ion current normalization and QC-based relative standard deviation filtering (threshold: 30%) minimized technical noise. Metabolite annotation was conducted against a custom MS2 spectral library, with a similarity score threshold of 0.3. Pathway enrichment analysis was performed using the KEGG database (https://www.kegg.jp/kegg/pathway.html, accessed on 15 March 2024).

### 2.6. Statistical Analysis

Data were analyzed using one-way analysis of variance (ANOVA) within SPSS Statistics 29.0; a value of *p* < 0.05 was considered statistically significant. The results were visualized using Origin 9.0. All data were expressed as the mean ± standard deviation (SD). Principal component analysis (PCA) and orthogonal partial least squares discriminant analysis (OPLS-DA) were performed using Simca-14.0 software [26].

## 3. Results and Discussion

### 3.1. Optimization of MF Intensity for PHA Biosynthesis

**Figure 2:**
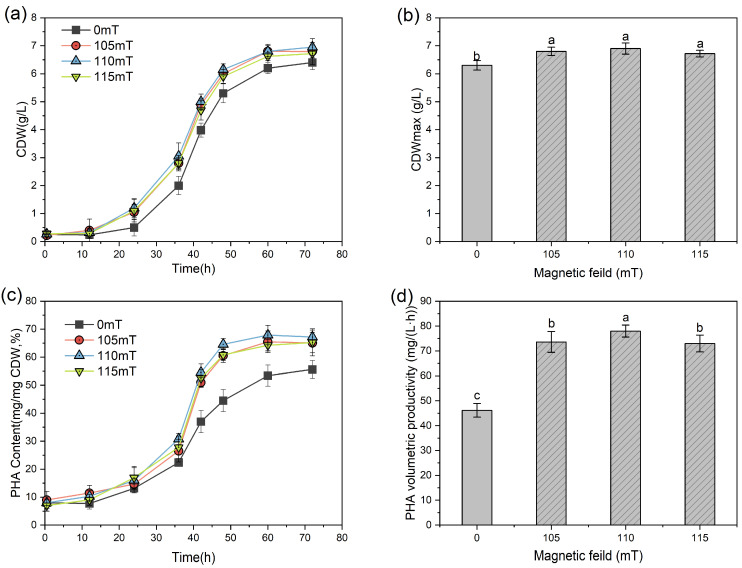
Growth and PHA synthesis of *H. mediterranei* under different SMF intensities. (**a**) CDW concentration, (**b**) CDW_max_ concentration, (**c**) PHA content, and (**d**) PHA volumetric productivity. For a, b, and c, different letters in the same column indicate statistically significant differences (*p* < 0.05).

Based on a previous identification of SMF windows for PHA synthesis in *H. mediterranei* [21], this study refined the MF intensity determination using an upgraded electromagnetic system with a lower MF gradient (5 mT). The results showed that all tested MF intensities (105–115 mT) increased cell density (*p* < 0.05). The maximum CDW concentration (CDW_max_) in the MF group reached 6.72 ± 0.12 to 6.88 ± 0.16 g/L compared to 6.29 ± 0.17 g/L for the control group. There were no significant inter-group differences among MF intensities for biomass accumulation (*p* > 0.05) (Figure 2a,b). The PHA synthesis profiles (Figure 2c) showed MF intensity-dependent accumulation patterns, with the 110 mT condition reaching peak cellular levels of 67.82 ± 1.81%. This level exceeded both the control group (*p* < 0.01) and 105/115 mT condition (*p* < 0.05) samples. The PHA volumetric productivity analysis verified this pattern, as the 110 mT group’s PHA volumetric productivity (77.97 ± 2.40 mg/(L·h)) exceeded both the control group (46.01 ± 2.87 mg/(L·h), *p* < 0.001) and samples with other intensities (72.64~73.12 ± 3.88 mg/(L·h), *p* < 0.05). These results conclusively identified 110 mT as the optimal MF intensity density for maximizing both biomass concentration and PHA levels in *H. mediterranei* cultures.

### 3.2. Comparative Impacts of MF Types on PHA Biosynthesis

**Figure 3:**
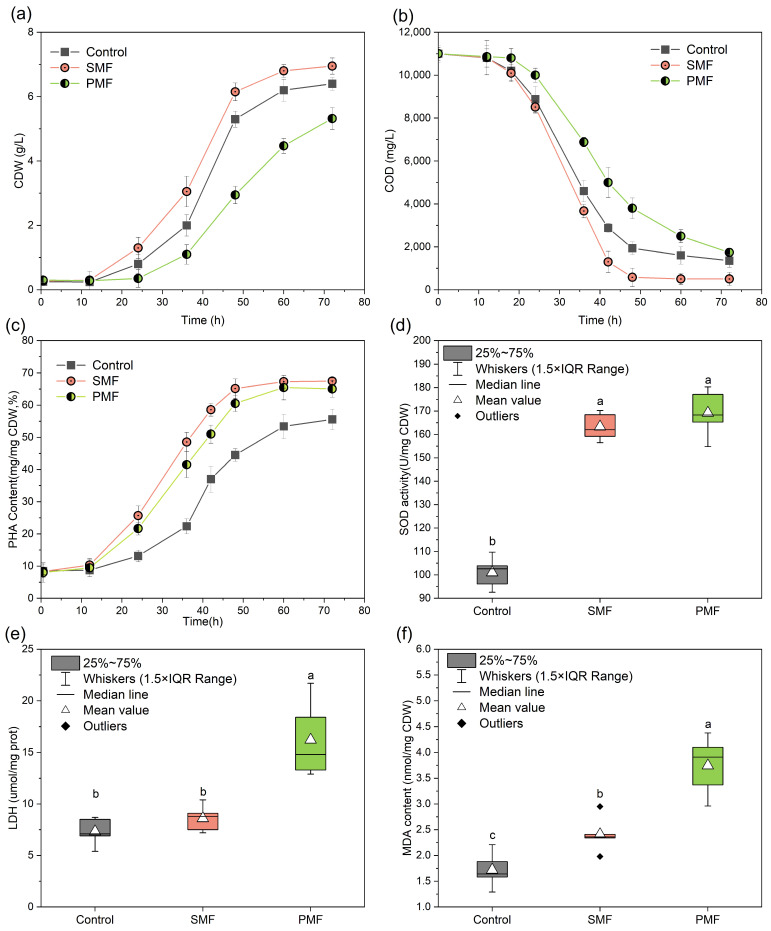
Growth, PHA synthesis, and enzyme activity of *H. mediterranei* under SMF and PMF conditions. (**a**) CDW concentration, (**b**) COD concentration, (**c**) PHA content, (**d**) SOD activity, (**e**) LDH release, and (**f**) MDA content. For a, b, and c, different letters in the same column indicate statistically significant differences (*p <* 0.05).

Building on the MF intensity optimization testing, SMF versus PMF methods were compared at 110 mT to further describe the magnetobiological mechanisms (Figure 3). The CDW analysis revealed diametrically opposed effects: the SMF increased biomass accumulation to 6.85 ± 0.25 g/L, which was higher compared to the control group. The PMF suppressed growth (5.32 ± 0.34 g/L, *p* < 0.05) with a prolonged lag phase (+12 h extension) (Figure 3a). The COD concentrations in the fermentation broth (Figure 3b) showed that the PMF inhibited substrate absorption by *H. mediterranei*. The glucose utilization rate for the 72 h testing duration was 84.18%, which was lower than the 95.45% result in the SMF group and the 87.73% result in the control group (*p <* 0.05). Despite impaired substrate uptake in response to the MF, the intracellular PHA levels reached 64.05 ± 2.59%. This significantly exceeded the controls (6%, *p <* 0.05) but remained below the SMF levels (68.14 ± 2.06%) (Figure 3c). This outcome indicates that PMF reduced the uptake of extracellular carbon sources, but facilitated intracellular metabolic redirection to PHA synthesis.

Studies have shown that the cellular uptake of substrates relates closely to the cell membrane structure and the membrane channels [27]. The intracellular activities or concentrations of LDH, MDA, and SOD were analyzed to characterize lipid peroxidation, membrane integrity, and antioxidant responses in *H. mediterranei* under MF exposure. LDH is exclusively localized in the cytoplasm; as such, its leakage indicates membrane structural damage caused by external environmental stress [28]. The results revealed that the extracellular LDH concentration in the PMF group (16.41 ± 6.34 umol/mg prot) increased by 1.2-fold compared to the control group (7.38 ± 1.86 μmol/mg prot) (Figure 3e). Thus, it was considered that PMF exposure disrupted membranes in *H. mediterranei*. These findings are align with the study by Qian et al. [14], where PMF-treated bacterial cells displayed increased surface roughness, compromised membrane integrity, and significant leakage of intracellular contents, further corroborating the above conclusions.

In general, when reactive oxygen species (ROS) levels increase in microbial cells, there is oxidative damage to lipids, particularly in polyunsaturated fatty acids. This leads to MDA formation through peroxidation [29]. Both MF treatment groups saw an elevated MDA content compared to the control, with the PMF group reaching 3.80 ± 0.70 nmol/mg CDW. This was 2.2-times higher than the control group (1.73 ± 0.51 nmol/mg CDW). PMF appeared to cause a significant ROS accumulation in *H. mediterranei*, which oxidized PUFA and then generated a large amount of MDA. SOD is a critical enzyme that defends against oxidative damage; its levels reflect the cellular capacity to scavenge free radicals [30]. Figure 3d shows that SOD activity significantly increased by 57.28% and 63.07% in SMF and PMF groups, respectively, compared to the controls (*p <* 0.05). The PMF triggered SOD upregulation, but it did not fully counteract ROS, leading to membrane damage. These results align with the study of magnetic-induced ROS generation [31], where PMF generated transient electromagnetic eddies that increased radical formation. More generally, the observed biological fragility under PMF drives its antimicrobial applications in food sterilization [32]. From a PHA-production perspective, SMF emerged as the optimal choice in this study, synergistically increasing both substrate conversion efficiency and PHA volumetric productivity without inducing cytotoxic stress.

### 3.3. Effect of MF Exposure Durations on PHA Synthesis

**Figure 4:**
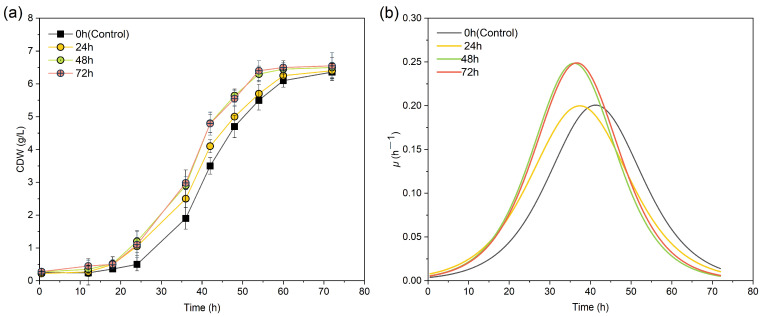
Growth of *H. mediterranei* under different SMF exposure durations. (**a**) CDW concentration, (**b**) specific growth rate (*μ*).

The duration of MF exposure also plays a critical role in magnetobiological effects, with Tomska et al. demonstrating that nitrification only increased when the sludge was exposed to sustained MF [33]. Figure 4a shows that all SMF-exposure groups experienced a superior CDW compared to the control during the first 24 h, effectively shortening the lag phase from 24 h (control) to 18 h. The 24 h exposure group showed gradual CDW reduction after magnetic withdrawal. This indicated there were transient biomass enhancement effects. Both 48 h and 72 h exposure groups reached a stationary phase earlier; there was no significant difference in the maximum CDW between them (*p* > 0.05).

The specific growth rate analysis (Figure 4b) showed that SMF exposure significantly accelerated microbial metabolism, shown by the earlier attainment of *μ*_max_ (*p <* 0.05). Further, the group exposed to the SMF specifically during the logarithmic growth phase (24–48 h) had the highest *μ*_max_, reaching 0.249 ± 0.015 h^−1^. This was significantly higher compared to the control group and the 24 h exposure group (0.199 ± 0.010 h^−1^) (*p <* 0.05, Table 1). This demonstrates that the metabolically active logarithmic phase responded the most to SMF stimulation. Khokhlova et al. also highlighted growth-stage-dependent MF effects [34]. As such, in terms of biomass accumulation, applying the SMF solely during the first 48 h saw nearly identical effects as full-cycle SMF exposure.

**Figure 5:**
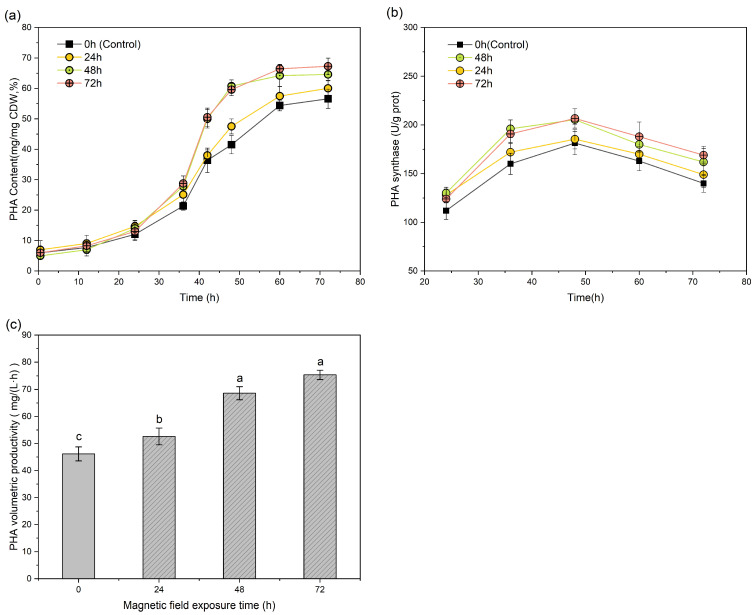
PHA production in *H. mediterranei* under different SMF exposure durations. (**a**) PHA content, (**b**) PHA synthase activity, (**c**) PHA volumetric productivity. For a, b, and c, different letters in the same column indicate statistically significant differences (*p <* 0.05).

For PHA biosynthesis (Figure 5a), prolonged SMF exposure had cumulative enhancement effects. The 72 h exposure group showed optimal PHA volumetric productivity (75.35 ± 1.74 mg/(L·h)), representing 63.3% and 12.3% increases over the control (*p <* 0.01) and 48 h exposure groups (*p <* 0.05), respectively. Enzyme activity tracking (Figure 5b) also had time-dependent magnetic effects: the 24 h exposure group showed a 14.3% lower level of PHA synthase activity than the 48 h group at 36 h (172.08 ± 4.67 vs. 196.16 ± 6.58 U/g prot, *p <* 0.05). This confirmed the reversible nature of magnetic stimulation on PHA synthase activity. These observations are consistent with Tao et al. [35], who reported transient increases and subsequent declines in microbial electrochemical performance following SMF removal.

The continuous 72 h exposure group reached the optimal PHA volumetric productivity (75.35 ± 1.74 mg/(L·h)), seeing 63.3% and 12.3% increases over the control (46.15 ± 2.59 mg/(L·h), *p <* 0.01) and 48 h exposure groups (67.08 ± 2.09 mg/(L·h), *p <* 0.05), respectively (Figure 5c). The group with 48 h exposure reached 90.11% of the maximum productivity seen with full-cycle exposure. Continuous SMF application throughout fermentation (72 h) is theoretically optimal for PHA volumetric productivity; however, practical engineering considerations indicate that 48 h exposure is a cost-effective strategy, as it significantly improves productivity with reduced energy input.

### 3.4. Metabolic Regulation Under SMF Exposure

**Figure 6:**
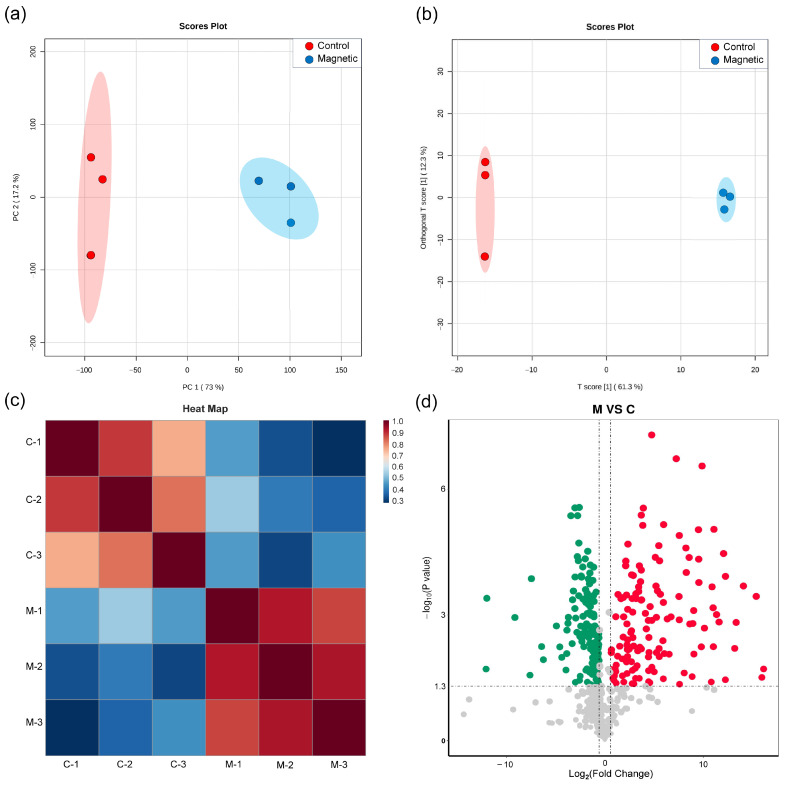
Preliminary analysis of metabolomic sample data. (**a**) Sample correlation analysis results from PCA. (**b**) Results from the OPLS-DA analysis. (**c**) Heat map of sample correlation analysis results (M: MF-treated group, C: control group); the numeric identifiers following the hyphen (e.g., M-1, C-3) correspond to individual sample labels. (**d**) Metabolomics volcano plot (each point represents a metabolite. Red point: upregulated. Green point: downregulated. Gray point: non-significant).

Metabolomics enables the systematic investigation of metabolic alterations in response to external stimuli by analyzing metabolites, which are the most fundamental phenotypic manifestations of organisms. After identifying the optimal SMF parameters for PHA synthesis in *H. mediterranei*, the metabolomics between the SMF and control groups were compared to describe the systematic metabolic regulation under SMF stimulation. PCA showed a clear separation between groups along PC1 (73.0% variance), significantly exceeding the intra-group variation (17.2%) (Figure 6a). OPLS-DA further confirmed distinct metabolic profiles; all samples were clustered within 95% confidence intervals (Hotelling’s T-squared ellipse, Figure 6b). Pearson correlation coefficients confirmed there was a high level of intra-group reproducibility (R^2^ > 0.8, Figure 6c). In general, the results show that the experimental reliability and sample selection were sufficient to support subsequent analysis.

The untargeted metabolomics identified 1858 metabolites, with 523 exhibiting significant alterations based on screening criteria of *p*-value < 0.05, Variable Importance in Projection (VIP) ≥ 1.0, and |log_2_FoldChange| > 0.58. These included 135 upregulated and 139 downregulated metabolites (Figure 6d). These differentially expressed metabolites spanned several core metabolic pathways: energy metabolism (e.g., glycolysis/gluconeogenesis, pyruvate metabolism), amino acid metabolism (e.g., amino acid biosynthesis, glycine, serine, and threonine metabolism), nucleic acid metabolism (e.g., pyrimidine/purine metabolism), and stress response processes (e.g., bacterial chemotaxis, glutathione metabolism).

**Figure 7:**
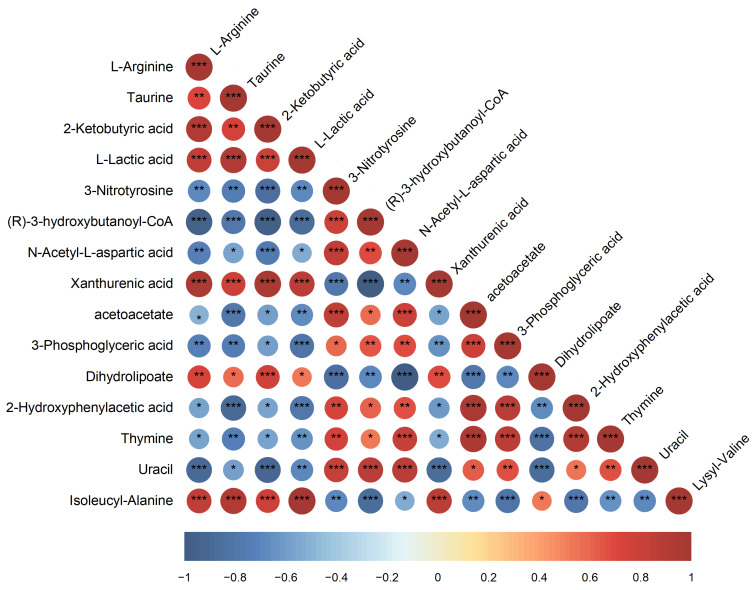
Heatmap of differential metabolite correlations. Top 15 metabolites ranked by VIP values from the OPLS-DA model; color scale indicates Pearson correlation coefficients, with red/blue representing strong positive/negative correlations, respectively (***: *p* < 0.001, **: *p* < 0.01, *: *p* < 0.05).

The top 15 differential metabolites screened using VIP scores from OPLS-DA models underwent a Spearman correlation analysis to further describe their interactions. Figure 7 shows robust positive correlations between taurine and (R)-3-hydroxybutanoyl-CoA, 3-nitrotyrosine, xanthurenic acid, and isoleucyl-alanine. Taurine and isoleucyl-alanine showed synergistic roles in promoting intracellular osmoregulation [36,37]. There was a strong positive correlation (*p <* 0.001) between 3-nitrotyrosine and xanthurenic acid. The substance 3-nitrotyrosine, a biomarker of oxidative stress, is generally associated with protein damage in *H. mediterranei* under high-salinity or radical-enriched conditions [38]. Xanthurenic acid, a metabolite derived from tryptophan, is generally implicated in oxidative stress responses. This association may arise from the role of tryptophan as a stress-related protein in archaea [39].

The PHA precursor (R)-3-hydroxybutanoyl-CoA was significantly positively correlated with metabolites involved in amino acid biosynthesis and carbon metabolism. Specifically, it was strongly associated with amino acid intermediates, including L-Arginine, taurine, 2-Ketobutyric acid, and N-acetyl-L-aspartic acid; it was also associated with the glycolytic intermediate 3-phosphoglyceric acid, which contributes to serine synthesis. These coordinated changes likely reflect an increased demand for energy metabolism and precursor supply during PHA biosynthesis. Further, (R)-3-hydroxybutanoyl-CoA was positively correlated with the pyrimidine catabolites thymine and uracil; their increased degradation provides carbon and nitrogen resources [40]; it was also correlated with Dihydrolipoate, a CoA-related molecule essential for pyruvate dehydrogenase-driven energy metabolism [41]. This network of interactions highlights the SMF’s regulatory role in promoting PHA accumulation. Conversely, (R)-3-hydroxybutanoyl-CoA was significantly negatively correlated with L-lactic acid, 3-nitrotyrosine, and acetoacetate. The strongest antagonism was seen with acetoacetate (r = −0.88, *p* < 0.05), indicating metabolic competition between PHA synthesis and acetoacetate-associated pathways.

**Figure 8:**
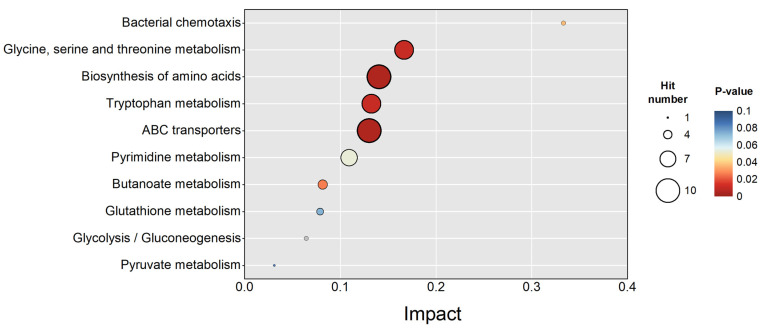
Top 10 significant enrichment pathways of metabolites. Hit number: the count of differential metabolites mapped to the pathway. Impact: the impact value of the topological analysis of the metabolic pathway.

Metabolic pathway enrichment analysis (Figure 8) shows that the most significantly enriched pathway was bacterial chemotaxis (Impact > 0.3, *p*-value < 0.01); this process depends on extracellular signal perception and transduction systems. As noted above, an SMF may influence the production of free radicals (such as ROS) to interfere with chemotactic regulation and metabolism. This general case is consistent with the study result indicating that both the SMF and the PMF stimulated intracellular ROS production. Concurrently, glutathione metabolism, which is a critical pathway for cellular antioxidant defense [39], showed a notable enrichment of differential metabolites. Specifically, ascorbic acid was upregulated by 12.94-fold (*p* = 0.0004). The enrichment of the ABC transporter pathway indicates an increased substrate uptake when exposed to SMF, corroborating the elevated COD degradation rates in the SMF-treated groups (Figure 3b). Different metabolites were also identified in energy metabolism pathways (glycolysis/gluconeogenesis and pyruvate metabolism); however, their enrichment levels were statistically insignificant (*p*-value > 0.05). This indicated that SMF primarily redirected carbon flux rather than amplifying overall energy production in this study.

**Figure 9:**
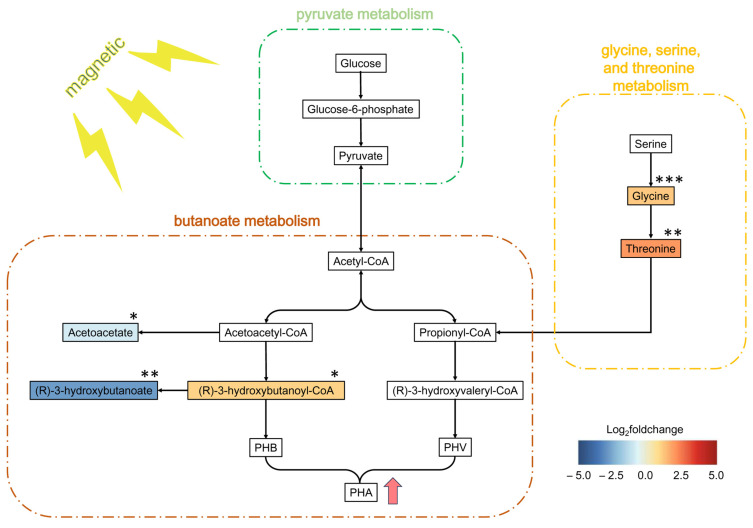
Schematic diagram of PHA biosynthesis pathways in *H. mediterranei*. Color gradients represent the log_2_(fold change) values of differential metabolites under SMF, with statistical significance denoted as follows: *** *p*-value < 0.001, ** *p*-value < 0.01, * *p*-value < 0.05.

Amino acid biosynthesis pathways were markedly enriched, potentially to meet the demand for energy metabolism and precursor synthesis [42]. Tryptophan, which is a precursor for membrane components, was significantly downregulated. This may have been due to ROS-induced membrane damage, requiring its consumption for repair. In contrast, threonine, glycine, and serine showed upregulation at a 2.1–5.8-fold level. These amino acids, along with malate and pyruvate, are potential biomarkers for polyhydroxyalkanoate (PHA) accumulation [42]. As illustrated in Figure 9, *H. mediterranei* inherently synthesizes propionyl-CoA (a precursor of poly-β-hydroxyvalerate, PHV) through acetyl-CoA conversion or metabolic routes involving serine, glycine, and threonine, bypassing valerate dependence. Increased threonine and glycine levels may further facilitate PHA synthesis through pathways involving glycine deamination and threonine degradation [43].

The butanoate metabolism pathway is closely linked to PHA synthesis and showed distinct metabolite alterations. The substance (R)-3-Hydroxybutanoyl-CoA was upregulated by 2.10-fold; (R)-3-hydroxybutanoate and acetoacetate decreased by 0.15- and 0.51-fold, respectively. Figure 9 shows that acetoacetyl-CoA was bifurcated into two branches. One branch was directed toward (R)-3-hydroxybutanoyl-CoA, which was then polymerized into poly-β-hydroxybutyrate (PHB). The other branch was directed toward acetoacetate formation though acetoacetate CoA-transferase. The SMF-induced downregulation of acetoacetate (0.51-fold) indicates there was a reduced flux toward this branch, favoring (R)-3-hydroxybutanoyl-CoA accumulation (2.10-fold upregulation) and subsequent PHB biosynthesis. The upregulation of (R)-3-hydroxybutyryl-CoA—the substrate for PhaC-mediated polymerization—suggests that an SMF enhances the enzymatic activity of *phaB* (acetoacetyl-CoA reductase) and *phaC* (PHA synthase). While our metabolomics data cannot directly address the issues at the gene or enzyme level, this speculation aligns with prior work showing that SMF upregulates *phaC* gene transcription by 3.23-fold in *H. mediterranei* [21]. The substance (R)-3-hydroxybutanoate is the depolymerized monomer of PHB. Concurrently, the decreased degradation of PHB to (R)-3-hydroxybutanoate (0.15-fold downregulation) further facilitated PHB accumulation.

## 4. Conclusions

This study systematically optimized MF parameters to increase PHA biosynthesis in *H. mediterranei*. The 110 mT SMF treatment maximized both biomass and PHA levels, outperforming the PMF treatment. The latter induced oxidative stress and membrane damage, despite increasing PHA synthesis. Prolonged SMF exposure (72 h) increased PHA levels by 63.3%. However, the 48 h exposure group reached a 90% efficiency level. This is a more practical and energy-saving approach. Metabolomics revealed there was an SMF-driven carbon flux redirection toward PHA synthesis by upregulating (R)-3-hydroxybutanoyl-CoA, glycine, and threonine, while downregulating (R)-3-hydroxybutanoate and acetoacetate. These results establish SMFs as a robust tool for increasing PHA volumetric productivity through metabolic regulation. However, scaling up SMF-assisted PHA production faces some challenges: generating uniform high-intensity SMF in large bioreactors is technically demanding, and the costs of SMF-generating equipment and energy consumption require rigorous cost–benefit analyses. Future studies could implement this magnetic-assisted strategy in cultures utilizing landfill leachate or industrial wastewater, to assess the impact of cost-effective PHA accumulation under SMF.

## Figures and Tables

**Table 1:** Effect of SMF exposure time on the kinetic growth parameters of *H. mediterranei* (media ± standard deviation).

Treatment Group	CDW_max_ (g/L)	T*μ*_max_ (h)	*μ*_max_ (h^−1^)	R^2^
Control	6.36 ± 0.26 ^a^	42.011 ± 0.288 ^a^	0.201 ± 0.006 ^b^	0.996
24 h	6.41 ± 0.71 ^a^	37.645 ± 0.421 ^b^	0.199 ± 0.010 ^b^	0.990
48 h	6.58 ± 0.32 ^a^	36.000 ± 0.326 ^b^	0.248 ± 0.013 ^a^	0.993
72 h	6.61 ± 0.46 ^a^	37.645 ± 0.423 ^b^	0.249 ± 0.015 ^a^	0.989

*μ*_max_ (maximum specific growth rate), T*μ*_max_ (time to reach the maximum specific growth rate of the sigmoidal curve), R^2^ (R-squared is the goodness-of-fit coefficient of the Gompertz model applied to the growth curve data). For a and b, different letters in the same column indicate statistically significant differences (*p <* 0.05).

## Data Availability

The original contributions presented in the study are included in the article, further inquiries can be directed to the corresponding author.

## Supplementary Materials

The following supporting information can be downloaded at https://www.mdpi.com/article/10.3390/polym17091190/s1, Table S1: Relationship between current and magnetic field intensity in the electromagnet device.

## Abbreviations

The following abbreviations are used in this manuscript:

*H. mediterranei*	*Haloferax mediterranei*
SMF	Static magnetic field
MF	Magnetic field
PMF	Pulsed magnetic field
PHA	Polyhydroxyalkanoates
PHB	Poly-β-hydroxybutyrate
PHV	Poly-β-hydroxyvalerate
COD	Chemical oxygen demand
CDW	Cell dry weight
SOD	Superoxide dismutase
MDA	Malondialdehyde
LDH	Lactate dehydrogenase
ROS	Reactive oxygen species
UHPLC	Ultra-high-performance liquid chromatography
DDA	Data-dependent acquisition
QC	Quality control
ANOVA	One-way analysis of variance
SD	Standard deviation
PCA	Principal component analysis
OPLS-DA	Orthogonal partial least squares discriminant analysis
OFAT	One-factor-at-a-time
VIP	Variable importance in projection
KEGG	Kyoto Encyclopedia of Genes and Genomes
