# Supplementary material for: Static Magnetic Field Increases Polyhydroxyalkanoates Biosynthesis in Haloferax mediterranei: Parameter Optimization and Mechanistic Insights from Metabolomics
Source: Polymers (Basel). 2025 Apr 27;17(9):1190. doi: 10.3390/polym17091190 (PMC12073411; doi:10.3390/polym17091190)
Supplement: Supplementary file 1 [file polymers-17-01190-s001.zip › polymers-3567661-supplementary.pdf]

Table S1. Relationship between current and magnetic field intensity in the electromagnet device.

Current（A）	Voltage（V）	Magnetic Field Intensity（Mt）
0	0	0 ± 0
1	2.4	10 ± 1
5	12	52 ± 2
10	24	106 ± 2
15	36	154 ± 3
20	48	201 ± 3
30	72	279 ± 3
